# Transformation and Sustainable Development of China’s Pig Industry in the Post-African Swine Fever Era: A One Health Perspective

**DOI:** 10.3390/vetsci13090960

**Published:** 2026-09-14

**Authors:** Ting Lin, Zengyong Zhu, Keyi Duan, Yakun Huang

**Affiliations:** 1 College of Economics and Management, China Agricultural University, No. 17 Qinghua East Road, Haidian District, Beijing 100083, China; linting20210301@163.com; 2 Institute of Animal Science, Chinese Academy of Agricultural Sciences, No. 2 Yuanmingyuan West Road, Haidian District, Beijing 100193, China; zhuzengyong@caas.cn (Z.Z.);

**Keywords:** African swine fever, One Health, pig industry transformation, biosecurity, sustainable livestock production

## Abstract

The 2018 African swine fever (ASF) outbreak disrupted China’s pig industry but spurred transformations in production, organisation, and governance. Using the One Health framework, this study examines post-ASF dynamics across animal health, environmental sustainability, public health, and economic sustainability. The findings indicate the following: (1) Policy, investment, biosecurity, and digital tech enabled rapid production recovery, yet the growth rate of production has significantly outpaced that of consumption, raising periodic overcapacity risks. (2) The shift from farrow-to-finish to specialised, large-scale operations improves efficiency and risk control but intensifies environmental pressure. (3) Digital tools and biosecurity promote precision and health-oriented farming. (4) Key sustainability challenges include crop–livestock decoupling, low manure utilisation, animal welfare gaps, and antimicrobial stewardship. The analysis reveals that while economic sustainability has driven rapid capacity recovery, it increasingly conflicts with environmental and animal health objectives. Therefore, the future pathways should adopt One Health to integrate capacity optimisation, green recycling, biosecurity governance, and digital transformation, balancing economic returns, animal health, environmental protection, and public health security.

## Simple Summary

The transition of China’s pig industry in the post-African swine fever era is an urgent issue for its modernisation. Using the One Health framework, this paper reviews the evolution, challenges, and future directions from four aspects: animal health, environmental sustainability, public health, and economic sustainability. The results show that policy, capital, biosecurity, and digitalisation drove rapid production recovery, but weaker consumption raises overcapacity risk. The farming model has shifted from farrow-to-finish to specialised large-scale operations, improving efficiency and resilience but increasing environmental pressure. Digital and biosecurity technologies promote precision health farming. However, crop–livestock decoupling, poor manure use, animal welfare gaps, and antimicrobial control issues remain barriers to sustainability. Future efforts should integrate the One Health approach to coordinate capacity optimisation, green recycling, biosecurity governance, and digital upgrading, promoting high-quality good farming practices for sustainable development.

## 1. Introduction

Pork is one of the most widely produced and consumed meat types globally, accounting for approximately one-third of total world meat production [1]. China is the world’s largest producer and consumer of pork; according to data from the National Bureau of Statistics of China, the country’s pork output reached 59.38 million tonnes in 2025 [2]. Based on Food and Agriculture Organization of the United Nations (FAO) data, China’s pork production accounts for about half of the global total [3]. The pig industry in China not only determines the global pork supply pattern but also influences world food security and the sustainable development of livestock production. In recent years, the concept of sustainable agriculture has gained increasing attention in the pig farming sector, emphasising the need to strike a balance between production capacity, environmental friendliness, animal welfare, and public health [4]. Against this backdrop, the transformation of China’s pig industry from a decentralised pattern dominated by smallholder farmers toward an industrialised, capital-intensive system presents both opportunities and challenges for sustainable agricultural development.

The ASF epidemic has had far-reaching impacts on the global pig industry [5]. This is particularly true for the outbreak that emerged in China in August 2018 and, as of 2026, has become a persistently endemic challenge [6], delivering an unprecedented shock to the country’s pig industry. Existing studies have primarily focused on three aspects: production, markets, and disease prevention and control. On the one hand, a substantial body of research has analysed the effects of the ASF outbreak on pig production capacity, pork prices, and international trade [7,8,9]. These studies have suggested that ASF led to a rapid decline in China’s hog inventory, with a year-on-year decrease of 27.5% by the end of August 2019 [10], triggering pork supply shortages and sharp price increases, and prompting the Chinese government to adopt production capacity restoration policies to facilitate industry recovery [11,12]. On the other hand, as the impact of the epidemic gradually subsided, some studies began to examine the long-term effects of ASF on China’s industrial structural adjustment; these studies have indicated that the ASF outbreak strengthened risk management awareness among farming entities and promoted the transition of the pig industry toward larger-scale, specialised, and high-biosecurity operations [13,14,15,16,17]. In recent years, with the deepening of the sustainable development concept, some studies have further focused on the environmental impacts and health governance issues of pig farming. Existing literature has pointed out that while large-scale farming improves production efficiency and disease prevention capabilities, it also poses environmental and public health challenges, such as manure resource utilisation, greenhouse gas emissions, and antimicrobial use [18,19]. In addition, some researchers have argued that, in the context of the persistent presence of major animal diseases, the One Health approach should be adopted to integrate animal health, ecological environment protection, and public health security, thereby achieving sustainable development of livestock production [20]. Overall, most existing studies have concentrated on the short-term effects of ASF on pork supply, prices, and farm losses, yet few have conducted in-depth analyses of the long-term structural changes in China’s pig farming industry after the ASF outbreak, particularly the interconnections among industrial upgrading, biosecurity, environmental sustainability, and economic resilience. In this paper, the post-ASF era refers to the period from the second half of 2019 to the present, which marks the end of the large-scale outbreak of ASF in China and the normalisation of the industry’s long-term coexistence of the epidemic and production. This term is used to refer to a new stage of development characterised by profound changes in industrial organisation, technology, and governance, rather than to imply the eradication of ASF.

The transformation of China’s pig industry cannot be evaluated solely from the perspectives of production capacity expansion and economic benefits. Increasing biosecurity risks, environmental pressures, and food safety concerns indicate that future livestock development needs to comprehensively consider animal health, ecological environment, and socio-economic factors [21]. The One Health framework offers an important analytical perspective for understanding the transformation of China’s pig industry in the post-ASF era, as it emphasises the interconnections among human, animal, and environmental health [20]. In the field of livestock production, One Health primarily involves three dimensions. The first is animal health, encompassing disease prevention and control, biosecurity management, veterinary care, and animal welfare, which directly affect production stability and efficiency. The second is environmental sustainability, focusing on manure resource utilisation, greenhouse gas emissions, pressure on soil and water resources, and the integration of crop and livestock production. The third is public health, relating to food safety, antimicrobial resistance, and risks of zoonotic diseases. However, relying solely on these dimensions is still insufficient to comprehensively evaluate the long-term sustainability of modern pig industry transformation. An economically unviable livestock sector cannot sustain long-term investments in biosecurity, environmental protection, or public health surveillance. Particularly in the post-ASF era, increased biosecurity investments, rising production costs, and heightened market volatility make economic sustainability a critical factor in measuring industrial resilience. Thus, economic sustainability is not an external add-on but an integral pillar that enables the other three dimensions to function effectively.

Therefore, building upon the One Health framework, this paper further introduces the perspective of economic sustainability and analyses the new developmental characteristics, practical challenges, and future pathways of China’s pig industry in the post-ASF era across four dimensions: animal health, environmental sustainability, public health, and economic sustainability. Specifically, this paper aims to address the following three questions: (i) What changes have occurred in China’s pig farming industry after the ASF outbreak? (ii) Has ASF driven the transformation and upgrading of China’s pig industry? (iii) Is this transformation sustainable? Accordingly, unlike existing studies that focus on the short-term supply shocks of ASF, this paper places emphasis on analysing how ASF has promoted the long-term transformation of China’s pig industry through enhanced biosecurity, technological upgrading, and industrial organisational adjustments, and evaluates its economic, environmental, and health implications within the One Health framework. The study is intended to contribute to the sustainable transformation of livestock production and to provide reference for policy design and industrial strategies in China’s pig sector.

## 2. Materials and Methods

This study adopts a qualitative synthesis approach, integrating data from multiple sources: (1) official statistics from the National Bureau of Statistics of the People’s Republic of China, the General Administration of Customs of the People’s Republic of China, the Ministry of Agriculture and Rural Affairs of the People’s Republic of China, Ministry of Ecology and Environment of the People’s Republic of China, FAO, the U.S. Food and Drug Administration, Brazilian Ministry of Agriculture, Livestock and Food Supply, and Brazilian Ministry of Health; (2) peer-reviewed literature indexed in Web of Science and CNKI; (3) industry reports and corporate financial disclosures.

**Figure 1:**
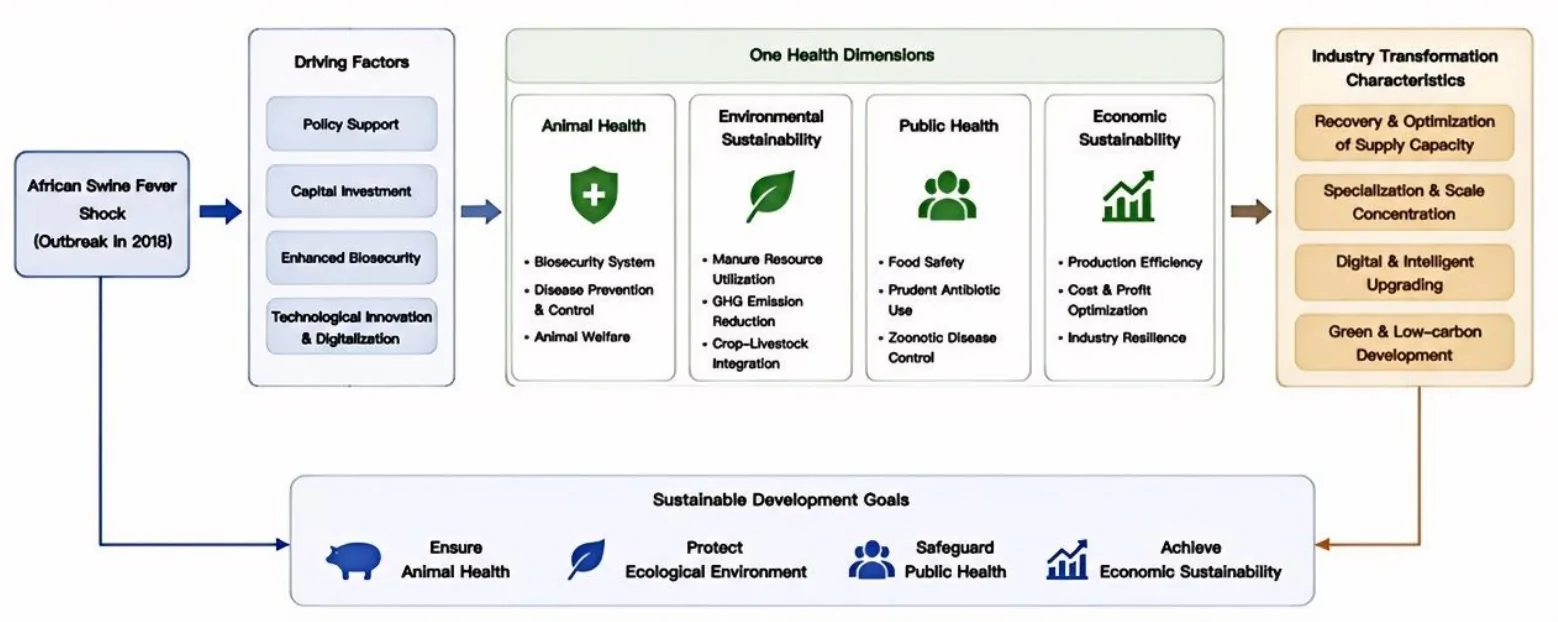
Analytical framework of China’s pig industry transformation after ASF under the One Health perspective.

The analytical framework operationalises the four One Health dimensions using the following indicators (Figure 1): animal health (biosecurity systems, disease prevention and control, animal welfare), environmental sustainability (manure resource utilisation, GHG emissions reduction, crop–livestock integration), public health (food safety, prudent antibiotic use, zoonotic disease control), and economic sustainability (production efficiency, cost and profit optimisation, industry resilience). Each transformation trend identified in Section 3 is mapped to these indicators in Section 4.

## 3. Transformation of China’s Pig Industry in the Post-ASF Era

Before the August 2018 ASF outbreak, China was already the world’s largest pork producer. In 2017, annual hog slaughter reached 702 million head, pork production totaled 54.52 million tonnes, and breeding sows stood at approximately 44.7 million [2]. The industry was predominantly small farmers, and farms with an annual pig production of less than 500 annual hogs supplied over 70% of the market [22]. However, the integration of China’s pig farming industry has been underway for over a decade. Between 2007 and 2017, the number of farmers decreased by 54.16% (from 82.35 million to 37.75 million), while farms (>500 heads) increased from 21.8% to 46.9% [22]. This indicates that post-ASF concentration trends were already in motion, albeit more slowly. Several structural challenges predated ASF. Environmental regulations since 2014 had forced smallholder exits via no-farming zone designations, contributing to declining inventories. In 2018, the industry was also in the downward phase of the hog cycle, with prices falling below costs and causing financial strain. Biosecurity vulnerabilities were widespread, especially among small and medium farms. Thus, environmental pressure, periodic decline, and structural fragmentation have laid the foundation for the rapid acceleration of the ASF epidemic’s transformation.

The ASF epidemic not only caused short-term disruptions to China’s pork supply system but also drove transformations in production organisation, technological systems, and governance models. In the post-ASF era, China’s pig industry has gradually shifted from the pre-epidemic decentralised production pattern toward a more large-scale, specialised, digitalised, and green-oriented trajectory. This transition has not only enhanced the industry’s supply capacity and production efficiency but has also altered the interrelationships among animal health, environmental sustainability, and economic resilience. The following sections analyse the transformation characteristics of China’s pig industry in the post-ASF era from four aspects: supply pattern, industrial organisational structure, production technology systems, and green production models.

### 3.1. Transformation of the Supply Pattern

In the post-ASF era, China’s pork market has moved beyond the initial stage of supply shortages that characterised the early outbreak period and has progressively entered a phase of relatively ample supply (defined here as production levels exceeding the normal-year benchmark of approximately 55 million tonnes, the reference level used by China’s Ministry of Agriculture and Rural Affairs for capacity planning [23]).

**Figure 2:**
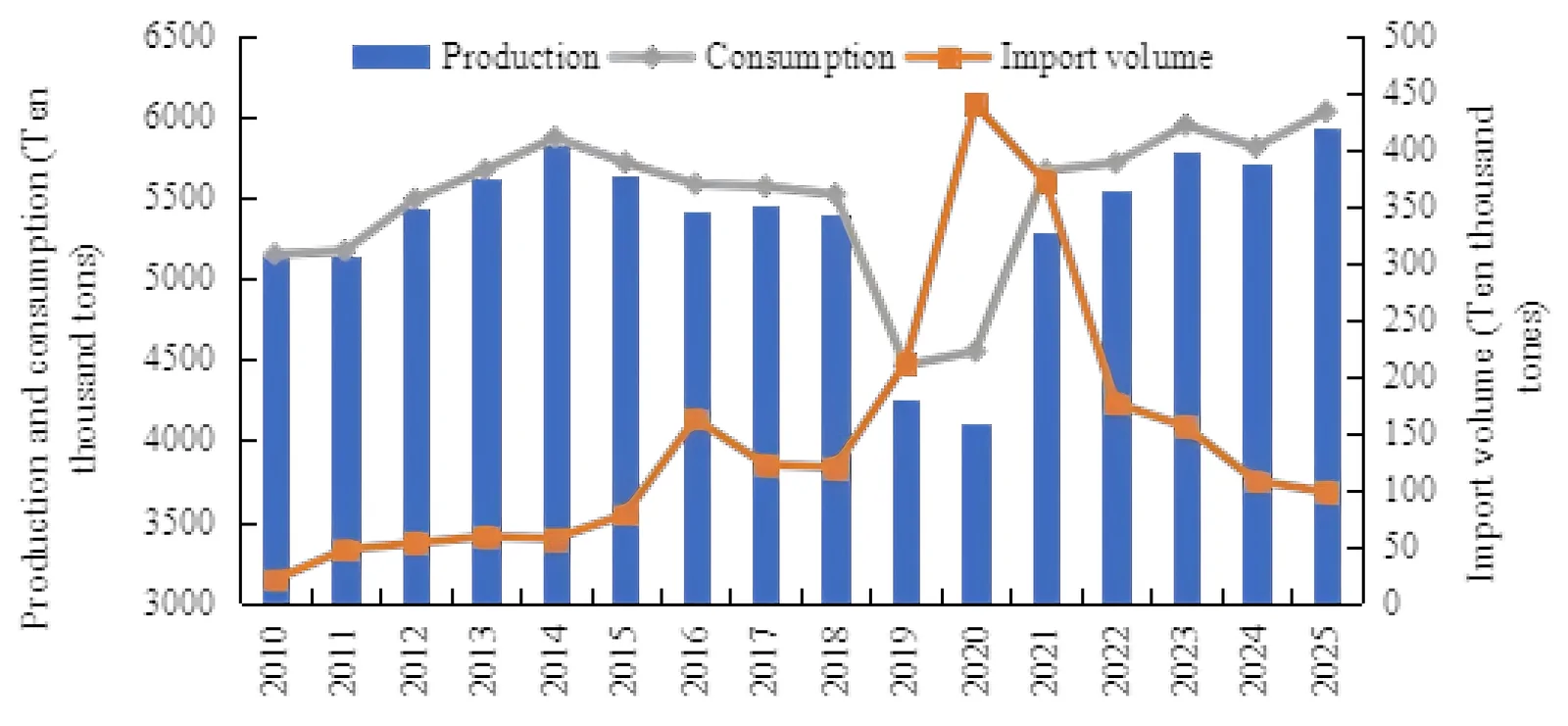
Trends in China’s Pork Production, Consumption, and Imports from 2010 to 2025. (Data source: obtained from the National Data database of the National Bureau of Statistics of China and the China Customs Database [2,26]).

From the supply side, the recovery of pig production capacity has been primarily attributable to the restoration of the breeding sow herd, policy support, and rapid capital influx. Following the massive culling of pigs in 2018–2019, China introduced sow subsidy programmes, financial support for large-scale farms and production capacity restoration policies to accelerate the repopulation process [24,25]. The recovery of breeding stock relied on both domestic retention and imports. After falling to a record low of approximately 1018 head in 2019 due to ASF-related border restrictions, imports of breeding sows rebounded to over 20,000 head in both 2020 and 2021 [26]. This two-phase strategy enabled China to restore both herd numbers and genetic quality, accelerating the supply recovery observed by 2025. Concurrently, the high pork prices prevailing after the ASF outbreak raised expected returns on industry investment, attracting substantial social capital into the pig sector. Leveraging advantages in capital, land, and technology, large enterprises expanded rapidly, significantly boosting the industry’s supply capacity. By 2025, China’s pork output had exceeded 59 million tonnes (Figure 2), surpassing the pre-ASF level [2]. The rapid recovery of supply capacity has altered the operational pattern of China’s pork market, yet it has also engendered new supply–demand imbalances. As pork consumption growth has stabilised while production capacity continues to expand, the industry has gradually shifted from under-supply to periodic overcapacity (defined here as production capacity consistently exceeding effective demand, with the breeding sow inventory remaining above the officially designated normal level (39 million head under the 2024 regulation, revised downward to 37.5 million head in 2026) for extended periods [23,27]).

From the demand side, China’s pork consumption has recovered steadily following the ASF-induced collapse in 2019–2020, but the pace of growth has moderated compared with the pre-ASF period. Per capita pork consumption rebounded from the trough of approximately 31.7 kg in 2019 to 40.5 kg in 2022 (surpassing the pre-ASF level), before stabilising at around 41–42 kg in 2023–2025. Meanwhile, poultry meat’s share of the total meat consumption has continued to rise, reaching 28.2% in 2025 compared with approximately 20–23% before 2018 [2,26], reflecting a gradual dietary shift toward diversified protein sources [28]. Overall, while absolute pork consumption has largely recovered to pre-ASF levels, its growth momentum has decelerated relative to the rapid expansion of production capacity.

From the import side, China’s pork imports surged during the ASF epidemic to alleviate domestic supply gaps. In 2020, China imported over 4.39 million tonnes of pork (Figure 2). The surge in pork imports in 2020–2021 was also consistent with the interruption of the global meat supply chain related to COVID-19, which has affected the trade flow of multiple livestock sectors. However, as domestic production capacity recovered, import dependence declined notably. By 2025, the proportion of pork imports (0.98 million tonnes, excluding pig offal) to total consumption (approximately 60 million tonnes) had fallen to 1.5% [2,26], indicating that the supply security capacity of China’s pig industry has been significantly strengthened.

Overall, in the post-ASF era, China’s pig industry has undergone a transition from supply scarcity and import supplementation to high self-sufficiency (defined here as the ratio of domestic production to total domestic consumption, with the official target set at approximately 95% [29]) and periodic surplus. The shift from supply scarcity to ample production affects multiple One Health dimensions. Economically, increased supply capacity enhances food security but introduces overcapacity risks that may erode farm profitability and reduce capacity for environmental and biosecurity investments. Environmentally, higher production intensifies manure generation and greenhouse gas emissions. Public health benefits from reduced reliance on imports but faces new challenges from intensive production systems. Thus, supply pattern transformation represents a trade-off among One Health dimensions that requires coordinated policy responses.

### 3.2. Transformation of Industrial Organisational Structure

The ASF epidemic has reshaped the traditional production organisation model of China’s pig industry, accelerating the development of a specialised division of labour within the industrial chain and the concentration of scale operations. For a long period, pig farming in China had been predominantly based on the farrow-to-finish model, in which sow breeding, piglet rearing, and finishing of market hogs were all integrated within the same farm. However, the ASF outbreak exposed the deficiencies of this traditional production model in terms of epidemic risk management. The concentration of sows, piglets, and finishing pigs within the same farm premises increases the risk of disease transmission and could lead to substantial economic losses. In the aftermath of the ASF epidemic, the specialised finishing model developed rapidly, and the stages of breeding, piglet production, and finishing have gradually been undertaken by different entities. Specialised division of labour reduces the complexity of production stages, thereby enabling risk isolation and improving production efficiency [30]. For example, finishing farms can focus on growth management, while breeding enterprises rely on genetic improvement and reproductive technologies to enhance piglet supply capacity. This vertical division of labour along the industrial chain has improved production organisation efficiency and also altered the profit distribution structure within the industry.

**Figure 3:**
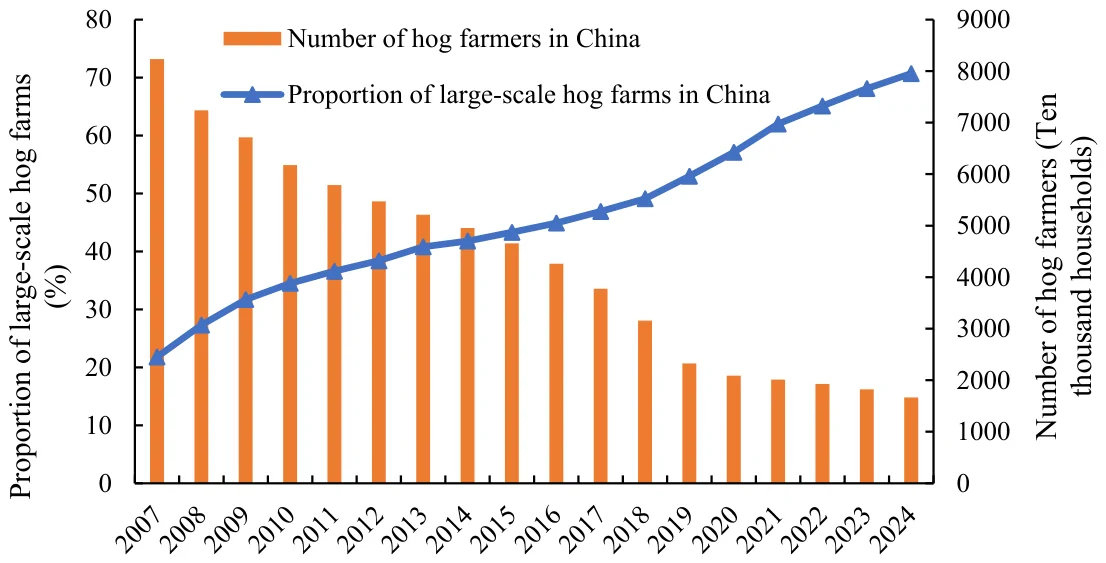
The trend in the proportion of large-scale hog farms and the number of hog farmers in China from 2007 to 2024. (Data source: compiled from the relevant annual editions of the China Animal Husbandry and Veterinary Yearbook [22]).

**Figure 4:**
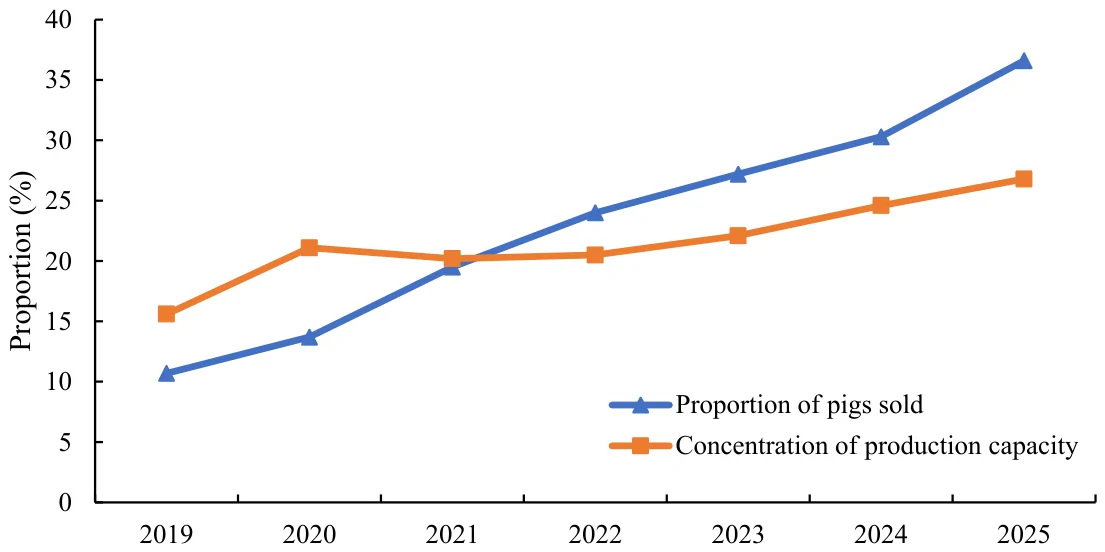
Trends in the proportion of pigs sold and production capacity concentration among the top 20 pig farming enterprises in China from 2019 to 2025. (Note: The 20 enterprises include Muyuan, Wens, New Hope, Zhengbang, and others (full list in Supplementary Table S1). Proportion of pigs sold refers to pig slaughter volume by these companies as a percentage of national total pig slaughter volume. Concentration of production capacity refers to breeding sows owned by these enterprises relative to the national breeding sow inventory. Data source: obtained from the National Data database of the National Bureau of Statistics of China and the publicly available financial data of Chinese pig farming enterprises [2]).

Concurrently, the ASF epidemic has further reinforced the trend toward scale concentration in the pig industry. The proportion of hog farms with an annual output of over 500 pigs in China increased from 49.1% in 2018 to 70.7% in 2024, and the number of hog farmers in China decreased from 31.56 million in 2018 to 16.66 million in 2024 (Figure 3). The concentration of production toward large-scale operations was already underway before 2018, with the share of farms (>500 heads) increasing from 21.8% in 2007 to 46.9% in 2017 (Figure 3). However, the ASF outbreak catalysed a step-change, as smallholders exiting the sector were disproportionately unable to meet new biosecurity requirements, while large enterprises, leveraging their advantages in capital, technology, and management, have expanded rapidly, achieving economies of scale in feed procurement, disease prevention and control, production management, and marketing [31]. By the end of 2025, the market hog shipments of the top 20 large pig enterprises accounted for 36.6% of China’s total pig shipments, an increase of 25.9 percentage points compared with 2019 (Figure 4). The development of large-scale operations has enhanced the industry’s risk resilience and facilitated the formation of a modernised production system [32].

This structural shift has mixed One Health implications. On the one hand, scale concentration has improved economic efficiency and pig health management through better biosecurity. On the other hand, high-density farming and regional concentration have exacerbated environmental carrying pressures and may increase the risk of localised disease transmission. Therefore, future optimisation of industrial organisation needs to strike a balance between scale efficiency and ecological carrying capacity.

### 3.3. Transformation of Production Technology Systems

The ASF epidemic has not only altered the mode of production organisation but has also driven the rapid upgrading of China’s pig farming technology systems. Among these developments, the establishment of biosecurity systems and the application of digital technologies have become critical factors underpinning the recovery of production capacity and the improvement of efficiency. First, the ASF outbreak significantly heightened the awareness of epidemic risk management among farming entities. In the post-epidemic era, pig farms have gradually shifted from a traditional disease-treatment model toward a full-process risk management approach, with increased investment in pen isolation, personnel and vehicle disinfection, all-in and all-out systems, batch production, and pathogen monitoring [33]. These measures have reduced the risk of disease transmission and improved herd health status and production stability [34]. Second, the accelerated application of digital technologies has further enhanced production efficiency. Intelligent environmental control systems, precision feeding equipment, automated management systems, and smart health monitoring technologies are being progressively integrated into the entire pig production process. By collecting real-time data on environment, feed intake, and behaviour, farming entities can adjust production management measures more precisely, thereby improving feed conversion efficiency and sow reproductive performance [35].

Unlike the gradual adoption of digital technologies in other sectors, the post-ASF digital transformation in pig farming was directly driven by the epidemic. Driven by the need for real-time health monitoring and contact tracing, immediate use cases were created that accelerated return on investment calculations for farmers. From the One Health perspective, digital technologies not only enhance economic efficiency but also improve animal health through early disease warning, precision management, and environmental regulation [36,37], thereby providing technical support for the prevention and control of public health risks.

### 3.4. Transformation Towards Green Production

In recent years, China has persistently promoted the resource utilisation of livestock and poultry manure, with continuous improvements in treatment facilities at large-scale farms, and the dissemination of technologies such as anaerobic digestion, composting, and nutrient recovery. The environmental pressures from manure concentration are primarily a consequence of scale expansion, which, as established above, was an ASF-accelerated rather than ASF-initiated trend. However, the policy push toward resource utilisation of manure predated ASF, with the Livestock and Poultry Manure Resource Utilization Action Plan (2017–2020) launched in 2017 [38]. The post-ASF period saw these policies intensified to address the environmental challenges created by rapid scale expansion. Thus, while ASF did not directly cause the green transition, it indirectly exacerbated the environmental problems that green policies are designed to solve, making the transition more urgent.

From the perspective of green development requirements, the transformation of the pig industry cannot rely solely on end-of-pipe pollution control, but needs to shift the production system from a ‘breeding-waste discharge’ model toward a circular model of ‘breeding-cropping-energy utilisation’ [39]. Therefore, the transition towards green production is not only an environmental governance imperative but also an important pathway for enhancing the industry’s long-term economic resilience and achieving sustainable development. This green transition directly addresses the environmental sustainability of One Health, while also contributing to public health by reducing antibiotic residues and airborne pollutants from manure management. Economically, however, the transition imposes significant upfront costs that may not be fully internalised by market returns, necessitating policy support and market-based mechanisms to ensure long-term viability.

## 4. Challenges Facing the Pig Industry in the Post-ASF Era and Pathways to Sustainable Transition

The foregoing analysis indicates that, in the post-ASF era, China’s pig industry has made notable progress in production capacity recovery, scale expansion, and production efficiency improvement. Nevertheless, the industrial transformation still confronts structural contradictions. From the One Health perspective, the current challenges pertain not only to the economic sustainability of the industry, but also to animal health, environmental health, and public health security. Imbalances between supply and demand arising from capacity expansion, environmental pressures resulting from the decoupling of crop and livestock production, and biosecurity and animal welfare issues in intensive farming, as well as technological and cost constraints in digital transformation, collectively constitute the main bottlenecks for the high-quality development of the pig industry in the future. Therefore, future industrial development needs to continue making breakthroughs in capacity regulation, green transition, health governance, and digital empowerment (Table 1).

### 4.1. Optimisation of Production Capacity Structure and Enhancement of Industrial Resilience

#### 4.1.1. Driving Factors for Rapid Recovery of Production Capacity

**Figure 5:**
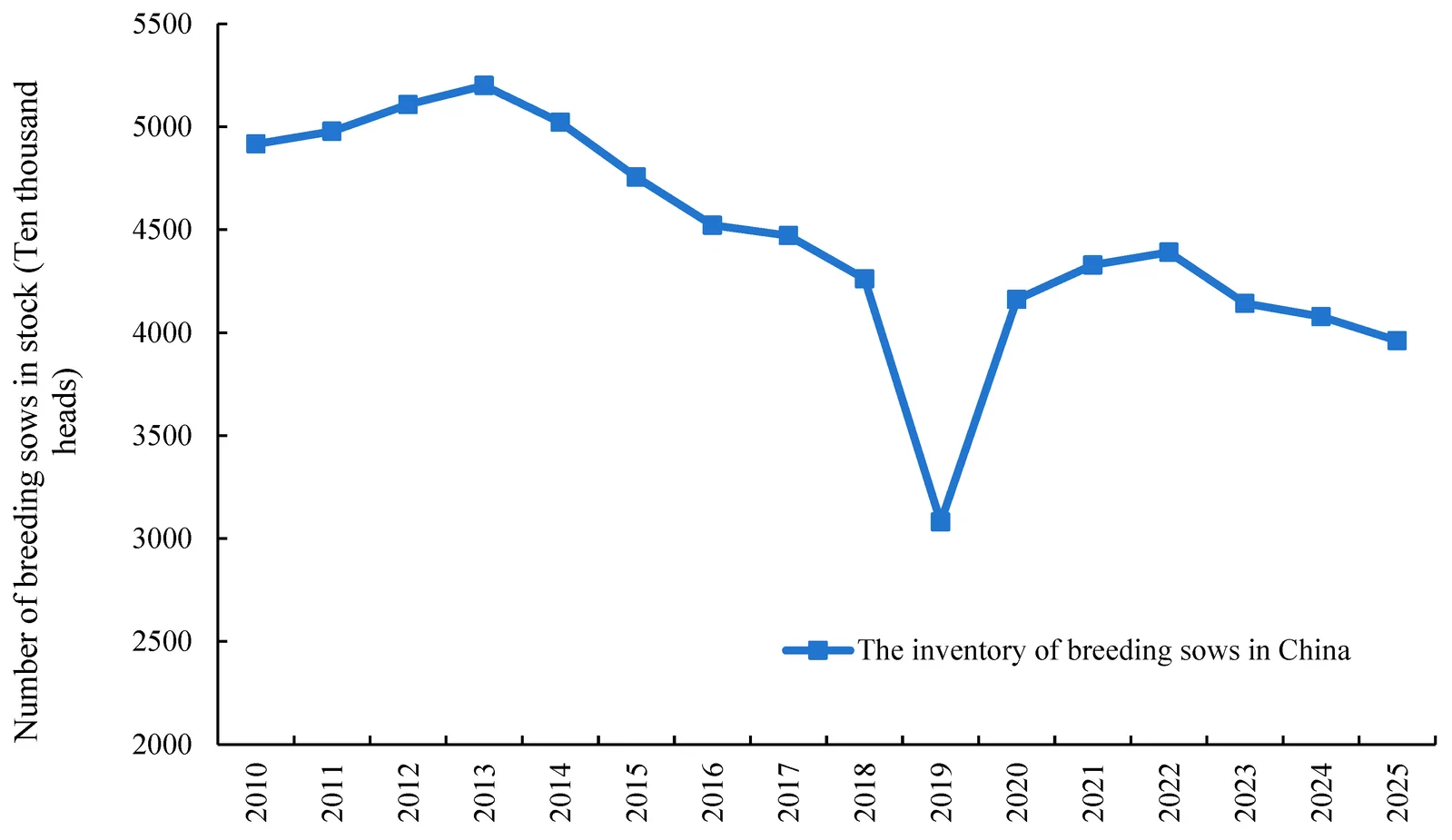
The trend of inventory of breeding sows in China from 2010 to 2025. (Data source: obtained from the National Data database of the National Bureau of Statistics of China [2]).

The high profitability following the ASF epidemic stimulated rapid expansion of pig production capacity, with capital inflows from large enterprises and restocking by small- and medium-sized farming entities jointly driving a significant increase in the industry’s supply capacity. Unlike traditional cyclical expansions, the growth in pig supply in the post-ASF era has stemmed not only from enlarged farming scales, but also from improved production efficiency and risk control capabilities. The prevention and control of ASF have promoted the refinement of biosecurity systems and the application of digital technologies, thereby enhancing herd health and production efficiency [40], allowing capacity recovery to shift from quantity-driven expansion to efficiency-driven growth, and further intensifying the pressure on supply growth. However, demand growth has moderated compared with the pace of supply expansion, resulting in gradually emerging overcapacity. By the end of 2025, the national breeding sow herd stood at 39.61 million head (Figure 5), still hovering near the upper bound of the normal reserve level. At the same time, sow reproductive efficiency continued to improve, with the national average pigs weaned per sow per year (PSY) reaching 23.4 in 2025, further augmenting the effective supply capacity [41].

There are other concurrent influences. The COVID-19 pandemic affected pork demand through reduced food service consumption, supply chain interruptions, and changes in consumer purchasing behaviour. Feed cost volatility, driven by global grain price fluctuations and international trade disruptions, also affected farm profitability independent of ASF. These factors compound the supply–demand imbalances and should be considered alongside ASF-related effects.

#### 4.1.2. The Consequences of Overcapacity

Under the dual effect of supply expansion and decelerating consumption growth, the industry has experienced a situation of increasing volume with declining prices. In 2025, the average annual pork price of China decreased by 15.2% year-on-year [42], and farming profits shrank markedly, with the profitability of some large enterprises declining [43]. For example, in 2025, the net profit attributable to shareholders of Muyuan (the largest pig producer in China) decreased by 13.29% year-on-year [44], while that of Wens (the second-largest pig producer in China) fell by 43.25% [45]. Moreover, the liability ratio of China’s pig industry remains high, with some enterprises exceeding 80%.

#### 4.1.3. Design of Capacity Regulation Mechanism

Overcapacity not only affects the economic stability of the industry, but may also weaken the capacity of farming entities to sustain investment in biosecurity, environmental governance, and technological upgrading, thereby reducing the industry’s long-term resilience. International comparisons offer relevant experiences. The EU manages dairy and grain production through quota systems [46], while the USA utilises crop insurance to stabilise farm incomes [47]. For China’s pig sector, a combination of dynamic sow inventory targets, production risk insurance, and temporary purchase schemes during severe price troughs could be considered. Smallholder exit strategies also warrant attention. The decline from 82.35 million farmers in 2007 to 16.66 million in 2024 raises questions about rural livelihoods [22]. Future policy should address transition support for exiting smallholders, including vocational training, alternative income opportunities, and compensation for sunk investments.

Consequently, the future pig industry needs to shift from quantity-driven expansion to quality-oriented improvement, and establish a capacity regulation mechanism oriented towards supply–demand matching, efficiency enhancement, and risk prevention. On the one hand, the regulatory targets for breeding sow inventories should be dynamically optimised based on changes in consumption demand and production efficiency, so as to avoid cyclical over-expansion. On the other hand, the capacity for biosecurity investment and the level of environmental governance should be incorporated into the industrial regulation evaluation system, thereby achieving the coordinated harmonisation of stable production, supply security, and sustainable development.

### 4.2. Insufficient Crop–Livestock Integration and the Construction of a Green and Low-Carbon Production System

#### 4.2.1. The Current Status and Bottlenecks of Fecal Resource Utilisation

The rapid development of large-scale farming has altered the traditional model of integrated crop–livestock production and has exacerbated the spatial mismatch between livestock production areas and cropland resources [48,49]. According to the Ministry of Agriculture and Rural Affairs, China’s livestock and poultry industry generates approximately 3 billion tonnes of manure annually [42]. Owing to the concentrated production of manure and the limited assimilative capacity of surrounding land, a large proportion of livestock waste cannot be recycled locally, leading to environmental risks such as nitrogen and phosphorus pollution, greenhouse gas emissions, and antibiotic residues [50,51]. Although the level of resource utilisation of livestock and poultry manure in China has been continuously improving in recent years, with the comprehensive utilisation rate of pig manure reaching 78% and the rate of treatment facilities equipped at large-scale farms reaching 98% [42], to date, China has allocated a cumulative total of RMB 22.052 billion in central government funds to support county-wide projects for manure resource utilisation in 305 counties, and pilot projects for green integrated crop–livestock circular agriculture in 337 counties (or farms) [42]. However, the integration of crop and livestock production remains constrained by factors such as land availability, insufficient coordination among stakeholders, lack of technical services, and limited economic returns.

#### 4.2.2. Greenhouse Gas Emissions and Carbon Footprint

Pig farming constitutes a significant source of agricultural greenhouse gas emissions. According to the National Greenhouse Gas Inventory Report, methane emissions from livestock manure management in China amounted to 110 million tonnes of CO_2_ equivalent in 2021, of which pig manure methane emissions accounted for approximately 70% [52]. The carbon footprint (CF) of pig farming in China ranges from 4.74 to 9.48 kg CO_2_ eq·kg^−1^, with an average value of 6.75 kg CO_2_ eq·kg^−1^, and this average value is generally higher than the data from large-scale pig farms in North America and Europe [53]. However, facilities for methane capture and utilisation from manure generally entail high construction investment and low returns, failing to generate effective economic incentives.

Carbon markets and emissions trading present potential solutions. In December 2025, China’s Ministry of Ecology and Environment, jointly with the Ministry of Agriculture and Rural Affairs, officially released the Methodology for Greenhouse Gas Voluntary Emission Reduction Projects—Biogas Recovery and Utilization from Manure in Large-Scale Pig Farms (CCER-15-001-V01). This methodology provides a standardised framework for large-scale pig farms to quantify and verify emission reductions from biogas capture and utilisation, enabling them to participate in China’s national voluntary emission reduction trading market and generate carbon credit revenues [54]. Subsequently, a complementary methodology for Centralized Treatment of Agricultural Waste was also issued, covering the mixed utilisation of livestock manure, crop straw, and vegetable waste, potentially unlocking the carbon asset value of China’s approximately 4 billion tonnes of annual agricultural waste [55].

With China’s national carbon trading market expanding, pig farms could generate carbon credits through methane capture and biogas production. However, the high upfront capital costs of anaerobic digestion and biogas facilities, typically requiring the investments of several million RMB per farm, coupled with the relatively low carbon price (survey data indicate that large-scale pig farmers’ average expected carbon price is approximately RMB 15.26/t CO_2_e [56]), limit broader participation. Blended finance mechanisms, combining government subsidies, carbon revenues, and private investment, could help overcome this barrier.

#### 4.2.3. International Experience Reference

The EU experience demonstrates that the core of the green transition in large-scale livestock production is not merely to enhance end-of-pipe treatment capacity, but to establish a Best Available Techniques (BAT) system covering the entire chain of feed, farming, manure treatment, and land application [57]. In Denmark, a study evaluating the costs of reducing ammonia emissions from finisher pig production using BAT found that techniques such as slurry cooling, acidification, and biofiltration can achieve substantial emission reductions at costs ranging from €2 to €10 per kg NH_3_ abated, depending on farm size and system configuration [58]. In England and Wales, case study analyses of three pig installations confirmed that the design and operation of animal housing and waste management systems are the most critical factors for successful BAT implementation, with significant environmental improvements achievable through ammonia emission reductions [59]. These diverse national experiences demonstrate that BAT implementation is not a one-size-fits-all approach but requires adaptation to local climate conditions, farm scales, and economic contexts. By integrating farming scale with land carrying capacity and nitrogen and phosphorus emission constraints, the EU has achieved a shift from end-of-pipe pollution control to life-cycle emission reduction [60]. Therefore, China can draw on these experiences by incorporating manure assimilation capacity into the planning constraints for large-scale farming, promoting the application of low-protein diets, precision nutrition, and resource recycling technologies, thereby enhancing the green competitiveness of the pig industry.

#### 4.2.4. From End-to-End Governance to Full Chain Management

With the implementation of the Ecological and Environmental Code of the People’s Republic of China, green development has become an important institutional guideline for the high-quality development of modern agriculture. The Code advocates adhering to green development and supporting high-quality development through high-level protection [52]. This implies that the green transformation of the pig industry should not merely focus on meeting pollution discharge standards, but should promote the transformation of production methods, resource utilisation patterns, and industrial organisation systems. For large-scale farming, manure management should shift from the traditional end-of-pipe treatment approach to a life-cycle management model covering feed utilisation, production process control, manure treatment, and resource recycling [61].

First, the incentive mechanism for crop–livestock integration should be improved, by providing support for agricultural facility land use, subsidies for manure resource utilisation, and the development of socialised service systems, so as to increase the participation motivation of farming entities. Second, the integration of manure resource utilisation with green and low-carbon development should be promoted. The core contradiction facing the green transformation of pig farming lies in the mismatch between environmental externalities and market returns. The resource utilisation of manure, the construction of carbon emission reduction facilities, and the transition to green production methods all require substantial upfront investment, yet their ecological benefits cannot be fully translated into market returns. Therefore, it is necessary to internalise environmental values through fiscal subsidies, green finance, and carbon market mechanisms, so as to transform green investment from a cost burden into an industrial competitive advantage. In the long run, establishing a circular agricultural system that integrates farming, cropping, and energy utilisation represents an important direction for achieving the environmental sustainability of the pig industry.

### 4.3. Health Governance Challenges and Cross-Sectoral Responses

#### 4.3.1. Animal Welfare Deficits and Their Productivity Trade-Offs

In the post-ASF era, while large-scale farming has improved production efficiency, it has also given rise to a series of challenges arising from the interplay among animal health, environmental health, and public health security. This has driven the governance of the pig industry to shift from a single-focus disease prevention and control approach toward a comprehensive One Health governance model encompassing animal health, food safety, and ecological risk management. Factors such as high-density housing, spatial confinement, transport stress, and shortened production cycles may compromise animal welfare standards and impair immune function and disease resistance in pig herds [62,63]. Chidgey (2024) found that space allowances below 0.34 m^2^ per pig (for pigs < 110 kg) significantly impair weight gain and feed conversion efficiency [62]. Animal welfare can also be assessed using indicators such as body condition scoring, lameness, skin tissue changes, tail and ear injuries, and stereotyped behaviour [64]. Therefore, animal welfare is not merely an ethical imperative but also a critical foundation for safeguarding animal health, reducing disease transmission risks, and improving production efficiency.

As China’s pork supply capacity increases, domestic consumption growth moderates, and the demand for international market expansion grows, competition in the pig industry will evolve from relying solely on output volume and cost advantages to encompassing product quality and health-oriented production capabilities. In the future, animal welfare standards should be enhanced through measures such as optimising stocking density, improving housing environments, and reducing production-related stress, while simultaneously meeting the non-tariff technical requirements of international markets concerning animal welfare, food safety, and environmental protection.

#### 4.3.2. Antimicrobial Resistance Risks and Stewardship Gaps

The inappropriate use of veterinary antimicrobials may promote the emergence of antimicrobial-resistant bacteria, which can subsequently affect public health through the food chain and environmental dissemination [65]. In large-scale farming systems, antimicrobials remain an important tool for disease treatment. However, prophylactic use, off-label application, and long-term low-dose administration may accelerate the development of resistance. Antimicrobial resistance has become a major global public health challenge, with transmission pathways including animal-derived foods, manure discharge, and environmental dispersal [66]. In July 2019, China’s Ministry of Agriculture and Rural Affairs issued Announcement No. 194, which set forth a phase-out plan for medicinal feed additives and adjustments to relevant regulatory policies, deciding to cease the production, importation, distribution, and use of certain medicinal feed additives and to revise the corresponding administrative measures [67]. Consequently, under the constraints of the legal and regulatory framework, reducing reliance on antibiotics, promoting precision diagnosis and treatment, and advancing alternative prevention and control technologies are not only an inevitable pathway for the modernisation of China’s livestock industry but also a key direction for achieving synergistic governance of animal health and public health.

The European Union (EU) has established a relatively stringent regulatory framework for antimicrobial governance, restricting group-level prophylactic use of antibiotics through veterinary pharmaceutical regulations and steering antimicrobial stewardship from drug-oriented control toward health-oriented management [68], providing valuable lessons for China’s pig industry in reducing antibiotic reliance and developing green pork products. Beyond the EU, the United States of America (USA) and Brazil have adopted distinct antimicrobial governance approaches. The USA has implemented the Veterinary Feed Directive (VFD) since 2017, requiring medically important antimicrobials in feed to be used only under veterinary supervision for therapeutic purposes, not growth promotion [69]. The U.S. Food and Drug Administration (FDA) Guidance #263 further transitioned over-the-counter antimicrobials to prescription status [70]. Brazil has shifted from permitting antibiotic growth promoters toward prohibition. Ministry of Agriculture, Livestock and Food Supply (MAPA) Ordinance SDA/MAPA No. 1.617/2026 banned the use of antimicrobials important for human or veterinary medicine as performance-enhancing feed additives [71], building on earlier restrictions including the 2016 colistin ban [72]. Brazil also established the National Action Plan for Antimicrobial Resistance (PAN-BR) through inter-ministerial collaboration [73].

China has established a national antimicrobial resistance surveillance system covering both human and animal sectors. However, integration between human and veterinary surveillance remains limited. Post-ASF restructuring of veterinary services, including expansion of official veterinarians and professionalisation of farm-level biosecurity officers, presents an opportunity to strengthen cross-sectoral AMR governance. For China’s pig industry, antimicrobial reduction is not only a requirement for safeguarding public health security but also a key pathway to enhancing the international competitiveness of pork. In the future, in order to access high-standard markets such as the EU, it will be necessary to progressively establish a residue control system covering the entire chain from feed, farming, and slaughtering to processing, and to upgrade pork products from merely safety-qualified to green, low-antibiotic, and biosecurity-certified standards. China should further improve its regulatory framework for antimicrobial reduction, explicitly prohibiting large-scale antibiotic use for prophylactic purposes, strengthening the supervision of antimicrobial contamination in feed, and reducing drug dependence through enhanced biosecurity levels and precision diagnostic capabilities.

#### 4.3.3. Pathways to Integrated One Health Governance

In the context of long-term ASF prevention and control, the establishment of compartmentalised disease-free zones, the refinement of biosecurity systems, and regionalised epidemic prevention models constitute important pathways for reducing the risk of major animal diseases. Meanwhile, as China’s pork supply capacity increases and market competition intensifies, expanding into international markets may become an important direction for alleviating China’s supply pressures. However, accessing high-standard markets such as the EU requires compliance with stringent animal health, food safety, and animal welfare requirements. The development of disease-free compartments not only reduces the risk of major animal diseases such as ASF but also serves as a fundamental basis for achieving international trade access. The EU has long relied on regionalisation and compartmentalisation systems to maintain safe trade from affected areas despite the presence of ASF risks.

In the future, further efforts should be made to translate the One Health concept from a theoretical framework into a governance system. China should improve its disease-free compartment and regionalised animal health management systems, drawing on the EU’s experience in risk-based zoning, and elevate disease-free compartments from internal biosecurity measures of individual enterprises to an institutional instrument linking disease prevention, trade facilitation, and international certification. Against the backdrop of persistent risks posed by major animal diseases such as ASF, safe trade under endemic conditions can be achieved through risk zoning, dynamic surveillance, and digital tracing, thereby enhancing industrial resilience and international market access capacity. At the same time, animal welfare standards should be strengthened and antimicrobial reduction management should be advanced, with the development of green, antibiotic-free and high-quality pork products that meet international market demands, using international standards as drivers to force industrial quality upgrading and achieve the synergistic development of economic sustainability with animal health and public health objectives. At the governance level, collaborative mechanisms should be fostered among agricultural and rural affairs, ecological environment, health and market regulation authorities, integrating animal health, environmental health and public health objectives into the evaluation system for pig industry policies, thereby transitioning from single-sector disease management to cross-sectoral health governance.

### 4.4. Digital-Driven Transformation Towards Precision Livestock Farming

#### 4.4.1. Application Scenarios and Documented Efficiency Gains

With the advancement of artificial intelligence, the Internet of Things, big data, and digital technologies, digitalisation is emerging as a key driver for improving the quality and efficiency of the pig industry. Technologies such as intelligent environmental control, precision feeding, health monitoring, and automated management can enhance production efficiency, potentially increasing PSY from 20 to over 30 and reducing labour costs by more than 50% [74], while also improving early disease warning and biosecurity management through real-time surveillance. For instance, some large enterprises such as Muyuan have already implemented digital management across the entire production process, with smart devices being widely applied in environmental control, precision feeding, and disease monitoring. The acceleration of digital technology adoption in pig farming was not solely ASF-driven. Broader trends, including China’s national Digital Agriculture Development Plan, advances in AI globally, and increasing labour costs, were creating favourable conditions for digital transformation regardless of ASF.

#### 4.4.2. Data Ownership and Privacy Concerns

Data ownership and privacy concerns represent an emerging governance challenge in the digital transformation of China’s pig industry. As farms increasingly adopt internet of things (IoT) sensors, AI monitoring systems, and cloud-based data platforms, vast amounts of production, health, and biosecurity data are generated and stored, raising questions about who owns, controls, and benefits from these data. Currently, farm-level data are often proprietary to technology vendors or large enterprises, creating the potential for data lock-in and limiting farmers’ ability to access or transfer their own production data. These concerns are compounded by the fact that China has yet to establish comprehensive, legally enforceable policies specifically addressing data ownership, privacy protection, and interoperability standards in the livestock sector. Future policy should prioritise the development of clear data ownership frameworks, interoperable data standards, and privacy-preserving infrastructure to build farmer trust and facilitate data sharing for public health surveillance and industry-wide efficiency gains.

#### 4.4.3. The Digital Divide Between Large and Small Farms

The digital divide between large and small farms constitutes one of the most pressing barriers to inclusive digital transformation. Recent data from 2025 reveal a stark disparity: while automation penetration among leading pig enterprises has exceeded 92%, the corresponding rate among small- and medium-sized farms is merely 18% [75]. This divide is driven by multiple factors. First, capital constraints are prohibitive. A basic intelligent feeding system costs approximately RMB 80,000, with annual data service fees of several thousand RMB, roughly equivalent to the entire annual profit of a small- or medium-sized farm [76]. Second, technical complexity poses barriers. Complex operating systems, incompatible data standards, and the need for specialised skills leave many smallholders unable to effectively deploy or maintain digital tools. Third, economies of scale favour large operations. The fixed costs of digital infrastructure can be amortised over larger production volumes, making the per-unit cost of digitalisation significantly lower for large enterprises than for small farms.

In response to these challenges, the industry has begun developing affordable, user-friendly tools such as smart collars, automatic weighing systems, and temperature sensors that address specific on-farm pain points without requiring full-scale infrastructure overhauls. Some technology providers have also shifted from selling hardware to offering equipment leasing models and data-as-a-service platforms [75], reducing upfront capital requirements. However, despite these innovations, the digital divide remains substantial. Addressing it will require coordinated efforts across multiple fronts: public investment in rural digital infrastructure, technical extension services tailored to smallholders’ needs, subsidies or tax incentives for technology adoption by small and medium farms, and the development of modular, interoperable digital tools that can be adopted incrementally.

#### 4.4.4. Potential Biases and Limitations in AI-Driven Health Monitoring

AI-driven health monitoring systems, while promising for early disease detection, are limited by biases introduced by data quality and representativeness. AI models trained predominantly on data from large commercial farms may not perform accurately on smallholder farms with different management practices or disease profiles [76]. Incomplete, inconsistent, or poorly documented sensor data further limit effectiveness [77]. To mitigate these risks, future development should prioritise the following: (1) rigorous model validation across diverse farm types; (2) human-in-the-loop approaches combining AI alerts with veterinary expertise; (3) transparent and interpretable models; and (4) continuous updating with farm-specific data.

#### 4.4.5. The Future Application of Digital Technology

In the future, it is necessary to promote the diffusion of precision livestock technologies from large enterprises to small- and medium-scale farms, and to strengthen the development of digital infrastructure and technical service systems [78]. The future direction of digital technologies should not be limited to improving production efficiency, but should also serve as a critical infrastructure connecting animal health, environmental management, and public health supervision. By establishing integrated systems for farming data, disease surveillance, input use, and product traceability, the pig industry can be steered from experience-based management toward a data-driven health-oriented production system.

## 5. Conclusions

### 5.1. Main Conclusions

The ASF epidemic has not only constituted a major external shock to China’s pig industry but has also served as a critical impetus for industrial restructuring and transformation of development models. Drawing on the One Health framework and incorporating economic sustainability, this paper analyses the transformation characteristics and sustainable development pathways of China’s pig industry in the post-ASF era, covering supply, production modes, governance, and future directions. The main conclusions are as follows:

- The analysis identifies four major transformations: pig supply shifting from scarcity to overcapacity; industrial structure shifting from farrow-to-finish to specialised large-scale farms; technology upgrading toward biosecurity and digitalisation; and green transition driven by scale-induced environmental pressures.
- ASF has driven this transformation, prompting farms to prioritise biosecurity and adopt specialised production. It has also accelerated large-scale expansion and digital technology adoption. However, scale expansion has intensified environmental pressures, making green transition more urgent. Policy changes, feed costs, dietary shifts, COVID-19 disruptions, and global technological advancements have further influenced this process.
- The analysis suggests these transformations have improved economic efficiency, biosecurity, and technological sophistication, all positive for sustainability. However, overcapacity risks, crop–livestock decoupling, environmental pressures, antimicrobial resistance, animal welfare gaps, and the digital divide threaten long-term viability.

The four dimensions of the One Health framework interact with one another in complex ways. Large-scale operations improve production efficiency and disease control capacity. However, rapid production expansion has intensified environmental pressures through the concentration of manure. Spatial planning that aligns farm location with land carrying capacity could mitigate this issue. At the same time, high-density stocking may compromise animal health. Conversely, biosecurity investments have enhanced production efficiency and generated positive economic returns, yet the high infrastructure costs of biosecurity create barriers for smallholders, for which tiered standards and targeted subsidies offer potential solutions. Furthermore, the benefits of specialised and digitalised farming systems may disproportionately favour large enterprises. Thus, public investment in shared digital infrastructure can promote more inclusive technology adoption.

### 5.2. Policy Implications

Therefore, future efforts should be guided by the One Health approach: improve the green circular system, promote the construction of disease-free compartments and health-oriented production models, strengthen animal welfare governance and antimicrobial reduction, and foster inclusive digital transformation to bridge the digital divide. This will help achieve synergistic enhancement of economic development, animal health, ecological environment, and public health security in the pig industry. Based on the analysis, we propose a phased roadmap:

- Phase 1 (2026–2027): establish dynamic capacity regulation mechanisms and expand AMR surveillance coverage.
- Phase 2 (2028–2030): implement spatial planning for manure management, pilot carbon trading for livestock operations, and develop open data standards for smart farming.
- Phase 3 (2031–2035): achieve full integration of One Health principles into pig industry policy, with cross-sectoral governance mechanisms linking agriculture, environment, and health authorities.

## Figures and Tables

**Table 1:** A systematic summary of transformation trends in and challenges to the four dimensions of One Health.

Transformation/Challenge	ASF-Related Mechanism	Evidence Type and Period	Animal Health	Environmental Sustainability	Public Health	Economic Sustainability	Key Policy Recommendation
Supply pattern shift	ASF supply shock → policy response + capital influx → rapid recovery → overcapacity	Official statistics; 2018–2025	○	●	●	●	Dynamic sow inventory targets; production insurance; import management
Industrial concentration	ASF accelerated pre-existing consolidation; smallholders unable to meet biosecurity standards	Statistical trend comparison; 2007–2024	●	●	●	●	Scale-ecological balance planning; regional disease containment; smallholder exit support
Technology upgrading	ASF created immediate demand for real-time health monitoring and biosecurity systems	Industry case studies; survey data; 2019–2025	●	○	●	●	R&D support; technology diffusion programmes; biosecurity certification
Green production	Scale expansion (ASF-accelerated) → intensified manure concentration → green transition urgency	Policy documents; environmental data; 2017–2025	○	●	○	○	BAT adoption; manure subsidies; carbon market integration
Overcapacity	Efficiency-driven growth + PSY improvement → supply outpaces demand	Official statistics; enterprise financial reports; 2019–2025	○	○	○	●	Dynamic sow inventory adjustment; production risk insurance; EU/US quota/crop insurance models
Crop–livestock decoupling	Scale concentration → spatial mismatch between production and land	Environmental monitoring; 2017–2025	○	●	○	○	Land-use planning; BAT framework; CCER carbon credits; blended finance
Health governance	Intensive farming → higher disease pressure + antimicrobial use	Literature review; policy documents; 2019–2026	●	○	●	○	AMR surveillance integration; welfare standards; veterinary service reform; compartmentalisation
Digital divide	ASF accelerated digital adoption in large farms, widening gap with SMEs	Industry survey; 2023–2025	○	○	○	●	Shared infrastructure; lightweight tools; equipment leasing; data standards

Note: ● = strong direct effect; ○ = moderate/indirect effect.

## Data Availability

The original contributions presented in this study are included in the article/Supplementary Material. Further inquiries can be directed to the corresponding author.

## Supplementary Materials

The following supporting information can be downloaded at: https://www.mdpi.com/article/10.3390/vetsci13090960/s1, Table S1: List of China’s top 20 pig farming enterprises; Tables S2–S5: Complete dataset for Figure 2, Figure 3, Figure 4 and Figure 5 with calculation notes.

## Abbreviations

The following abbreviations are used in this manuscript:

ASF	African swine fever
FAO	Food and Agriculture Organization of the United Nations
BAT	Best Available Techniques
PSY	Pigs weaned per sow per year
CF	Carbon footprint
EU	European Union
USA	The United States of America
VFD	Veterinary Feed Directive
FDA	U.S. Food and Drug Administration
MAPA	Ministry of Agriculture, Livestock and Food Supply
PAN-BR	The National Action Plan for Antimicrobial Resistance
IoT	Internet of Things
