# Supplementary material for: Transformation and Sustainable Development of China’s Pig Industry in the Post-African Swine Fever Era: A One Health Perspective
Source: Vet Sci. 2026 Sep 14;13(9):960. doi: 10.3390/vetsci13090960 (PMC13612062; doi:10.3390/vetsci13090960)
Supplement: Supplementary file 1 [file vetsci-13-00960-s001.zip › vetsci-4526608-supplementary.pdf]

Table S1. Top 20 Pig Farming Enterprises in China (Ranking Comparison: 2019 vs 2025).

Rank	2019	2025
1	Wens Foodstuff Group Co., Ltd.	Muyuan Foodstuff Group Co., Ltd.
2	Muyuan Foodstuff Group Co., Ltd.	Wens Foodstuff Group Co., Ltd.
3	Jiangxi Zhengbang Technology Co., Ltd.	Twins Group Co., Ltd.
4	New Hope Liuhe Co., Ltd.	New Hope Liuhe Co., Ltd.
5	Tech-bank Food Co., Ltd.	Zhengda Investment Co., Ltd.
6	COFCO Meat Investment Co., Ltd.	Sichuan Dekang Agriculture and Animal Husbandry Food Group Co., Ltd.
7	Twins Group Co., Ltd.	Jiangxi Zhengbang Technology Co., Ltd.
8	Guangxi Yangxiang Group Co., Ltd.	Tech-bank Food Co., Ltd.
9	Beijing Dabeinong Technology Group Co., Ltd.	Guangdong Haid Group Co., Ltd.
10	Sichuan Dekang Agriculture and Animal Husbandry Food Group Co., Ltd.	COFCO Joycome Foods Co., Ltd.
11	Hubei Xiangda Industrial Group Co., Ltd.	Guangxi Yangxiang Group Co., Ltd.
12	Shanxi Jinxiu Daxiang Agricultural and Animal Husbandry Group Co., Ltd.	Hunan New Wellful Co., Ltd.
13	Hunan New Wellful Co., Ltd.	Tangrenshen Group Co., Ltd.
14	Tiankang Biological Co., Ltd.	Guangxi Guiken Animal Husbandry Co., Ltd.
15	Tangrenshen Group Co., Ltd.	Guilin Liyuan Grain and Oil Food Group Co., Ltd.
16	Sichuan Tieqi Lishi Animal Husbandry Technology Co., Ltd.	Leshan Juxing Agricultural and Animal Husbandry Co., Ltd.
17	Guangxi Nongken Yongxin Livestock Group Co., Ltd.	Beijing Dabeinong Technology Group Co., Ltd.
18	Sichuan Tianzow Pig Industry Co., Ltd.	Shanxi Jinxiu Daxiang Agricultural and Animal Husbandry Group Co., Ltd.
19	Fujian Aonong Biological Technology Group Co., Ltd.	East Hope Group Co., Ltd.
20	Wuhan Jiangxia Jinlong Livestock and Poultry Co., Ltd.	Tiankang Biological Co., Ltd.

Note: Company names are translated based on common official or widely used English designations; where no official name was available, a standard transliteration or descriptive translation is provided.

Table S2. Trends in China's Pork Production, Consumption, and Imports from 2010 to 2025.

Year	Production	Consumption	Import volume
2010	5138.4	5147.6	20.1
2011	5131.7	5170.3	46.8
2012	5443.6	5489.1	52.2
2013	5618.6	5669.6	58.3
2014	5820.8	5868.1	56.4
2015	5645.4	5715.9	77.8
2016	5425.5	5582.6	162.0

2017	5451.8	5568.3	121.7
2018	5403.7	5518.8	119.3
2019	4255.3	4463.5	210.8
2020	4113.3	4551.4	439.1
2021	5296.0	5665.1	370.9
2022	5541.0	5714.0	175.7
2023	5794.0	5946.4	155.1
2024	5706.0	5810.5	107.2
2025	5937.8	6030.8	97.6

Note: Pork production data are sourced from the National Bureau of Statistics of China; Pork consumption is calculated as pork production plus pork imports minus pork exports (excluding pork by-products); Pork import volumes are drawn from the China Customs Database (excluding pork by-products).

Table S3. The trend of the proportion of large-scale hog farms and the number of hog farmers in China from 2007 to 2024.

Year	Proportion of large-scale hog farms in China	Number of hog farmers in China
2007	21.8	8235
2008	27.3	7238
2009	31.7	6714
2010	34.5	6174
2011	36.6	5787
2012	38.4	5470
2013	40.8	5212
2014	41.8	4954
2015	43.3	4656
2016	44.9	4261
2017	46.9	3775
2018	49.1	3156
2019	53	2327
2020	57.1	2088
2021	62	2010
2022	65.1	1926
2023	68.1	1823
2024	70.7	1710

Data source: compiled from the relevant annual editions of the China Animal Husbandry and Veterinary Yearbook.

Table S4. Trends in the proportion of pigs sold and production capacity concentration among the top 20 pig farming enterprises in China from 2019 to 2025.

Year	Proportion of pigs sold	Concentration of production capacity
2019	10.7	15.6
2020	13.7	21.1
2021	19.5	20.2
2022	24.0	20.5

2023	27.2	22.1
2024	30.3	24.6
2025	36.6	26.8

Note: The 20 enterprises include Muyuan, Wens, New Hope, Zhengbang, and others (full list in Supplementary Table S1). Proportion of pigs sold refers to pig slaughter volume by these companies as a percentage of national total pig slaughter volume. Concentration of production capacity refers to breeding sows owned by these enterprises relative to the national breeding sow inventory. Data source: obtained from the National Data database of the National Bureau of Statistics of China and the publicly available financial data of Chinese pig farming enterprises.

Table S5. The trend of inventory of breeding sows in China from 2010 to 2025.

**Year**	**The inventory of breeding sows in China**
2010	4916.4
2011	4977.6
2012	5108.0
2013	5200.6
2014	5021.9
2015	4755.4
2016	4521.7
2017	4471.5
2018	4261.0
2019	3080.5
2020	4161.3
2021	4328.7
2022	4390.5
2023	4142.1
2024	4077.6
2025	3961.0

Data source: obtained from the National Data database of the National Bureau of Statistics of China.
